# Acoustic scene classification based on three-dimensional multi-channel feature-correlated deep learning networks

**DOI:** 10.1038/s41598-022-17863-z

**Published:** 2022-08-12

**Authors:** Yuanyuan Qu, Xuesheng Li, Zhiliang Qin, Qidong Lu

**Affiliations:** 1 Weihai Beiyang Electrical Group Co., Ltd, Weihai, Shandong China; 2 School of Mechanical, Electrical, and Information Engineering, Shandong University, Jinan, China

**Keywords:** Electrical and electronic engineering, Environmental impact

## Abstract

As an effective approach to perceive environments, acoustic scene classification (ASC) has received considerable attention in the past few years. Generally, ASC is deemed a challenging task due to subtle differences between various classes of environmental sounds. In this paper, we propose a novel approach to perform accurate classification based on the aggregation of spatial–temporal features extracted from a multi-branch three-dimensional (3D) convolution neural network (CNN) model. The novelties of this paper are as follows. First, we form multiple frequency-domain representations of signals by fully utilizing expert knowledge on acoustics and discrete wavelet transformations (DWT). Secondly, we propose a novel 3D CNN architecture featuring residual connections and squeeze-and-excitation attentions (3D-SE-ResNet) to effectively capture both long-term and short-term correlations inherent in environmental sounds. Thirdly, an auxiliary supervised branch based on the chromatogram of the original signal is incorporated in the proposed architecture to alleviate overfitting risks by providing supplementary information to the model. The performance of the proposed multi-input multi-feature 3D-CNN architecture is numerically evaluated on a typical large-scale dataset in the 2019 IEEE AASP Challenge on Detection and Classification of Acoustic Scenes and Events (DCASE 2019) and is shown to obtain noticeable performance gains over the state-of-the-art methods in the literature.

## Introduction

Sounds contain a large amount of information about environments and form a major approach for humans to perceive the world. As a key sub-task of sound analysis, acoustic scene classification (ASC) has received significant attentions and made remarkable breakthroughs in theories and applications over the past few years. To classify an audio signal into one of the predefined classes that characterizes the environment in which it is recorded, ASC is widely used in various applications including machine hearing^1^, smart home^2^, scene monitoring^3^, and biological signal analysis^4^. For instance, a monitoring system reports an emergency by detecting abnormal sounds in a surveillance region. Similarly, a robot recognizes anomalies in environmental sounds and triggers necessary actions to interfere^5^. On the other hand, typical scenes (e.g., supermarkets or subway stations) feature sounds that are overlapped with each other and are possibly severely distorted by ambient noises. As compared with automatic speech recognitions (ASR), differentiating between environmental sounds poses a great challenge due to inter-class similarities and the lack of coherence in data, e.g., sounds from air-conditioning is hardly discernible from that of engine-idling when recorded in a distance.

From the perspective of algorithm developments, extracting domain-specific acoustic features as the first step is of critical importance to designing an algorithm with acceptable classification accuracies. Features in the time-domain and frequency-domain can be generated based on signal waveforms, e.g., Short-Time Fourier Transforms (STFT)^6^, Mel-Scale (Mel) Spectrogram^7^, Mel-Scale Frequency Cepstral Coefficients (MFCC)^8^, Constant-Q Transform (CQT)^9^ and various one-dimensional (1D) spectral properties. Traditionally, statistical models are used in classification tasks, such as Hidden Markov Model (HMMs)^10^, Gaussian Mixture Model (GMM)^11, 12^, and Support Vector Machines (SVM)^13,14^.

With the availability of large-scale datasets due to the vast amount of audio clips recorded by mobile devices, statistical models are gradually replaced by deep-learning methods, which are generally based on convolution neural networks (CNN) and are shown to achieve much better performance in the ASC task. In^15,16^, the authors summarized the performance of various deep-learning backbone models as typically used in computer visions, e.g., VGG^17^, Xception^18^, ResNet^19^, recurrent neural networks (RNNs)^20^, and long-short-term memory networks (LSTM)^21^, and confirmed that performance superiorities over statistical approaches can be well generalized to acoustic scenes. Moreover, it is shown in^22–24^ that the so-called ensembled-learning approach based on the aggregation of several CNN models effectively improves performance as compared with a single deep-learning model due to the diversities of extracted features and system architectures^25,26^. In^27,28^, various data augmentation techniques in the time domain are applied to supplement training samples and alleviate overfitting risks, e.g., adopting multiple sampling rates, harmonic percussive source separation (HPSS), foreground–background sound separation (FBSS). Alternatively, a frequency-domain augmentation approach is conducted on the signal spectrogram, which can be converted to a two-dimensional (2D) heatmap provided pixel values are properly normalized^29–32^.

In this paper, we seek to leverage the diversities of signal features and exploit spatial–temporal correlations inherent in environmental sounds. Specifically, we make the following contributions. First, we propose a novel model to learn multi-domain discriminant features, which fuses learned deep features with chromatogram statistics derived from raw signals. Secondly, in order to utilize intrinsic dependencies in acoustic data, we introduce the concept of high-dimensional time frames by extending the convolution kernel to the time domain and build a three-dimensional (3D) deep neural network structure. The 3D convolution operations are performed on a stack of spectrogram images so as to learn contextual information on time frames. To increase the representation capacity and assist in the model training, we incorporate residual connections and squeeze-and-excitation (SE) channelwise attention mechanisms between convolution layers. The proposed model, which is termed the Multi-Input 3D-SE-ResNet, is evaluated on a typical large-scale acoustic scene dataset, i.e., the DCASE2019 dataset, and is shown to achieve noticeable performance gains.

The rest of the paper is organized as follows. In Section “Signal features”, we use the discrete-wavelet-transform (DWT) as a signal pre-processing technique and obtain multiple formats of frequency-domain features as the input to the model. In section “Multi-input 3D CNN model”, the proposed multi-input 3D CNN model is presented, which characterizes feature aggregations and attention mechanisms to capture correlations embedded in environmental sounds. In section“Numerical experiments”, the proposed model is evaluated on the DCASE2019 dataset. Finally, the conclusion is drawn in section “Conclusion”.

## Signal features

In this section, we first present an approach to perform signal pre-processing based on the DWT technique, and proceed to extract signal features from the frequency-domain representations of signals, e.g., STFT, Mel spectrogram, and chromatogram. Following that, extensive data augmentations are applied on the spectrograms to improve the robustness and performance of the proposed model.

### DWT

Considering that acoustic signals are usually recorded in dynamic environments, we adopt the DWT technique to mitigate the effects of ambient noises and eliminating artefacts introduced into spectrograms. For the DWT, the set of wavelet functions is derived from an initial wavelet *h(t)*, which is extended by a parameter *a* = 2^* m*^ and translated by a constant *b* = *k*2^*m*^. Consequently, the overall transform is given by (1)^33, 34^, where the integer values of *m* and *k* are defined by an analytical expression,

1 $$  \left\{ {\begin{array}{*{20}l}    {h_{{m,k}} (t) = \frac{1}{{\sqrt a }}h\left( {\frac{{t - b}}{a}} \right) = \frac{1}{{\sqrt {2^{m} } }}h\left( {2^{{ - m}} t - k} \right)} \hfill  \\    {WT_{f} (m,k) = \int_{R} {f(t) \cdot \overline{{h_{{m,k}} (t)}} } dt} \hfill  \\   \end{array} } \right. $$

**Figure 1:**
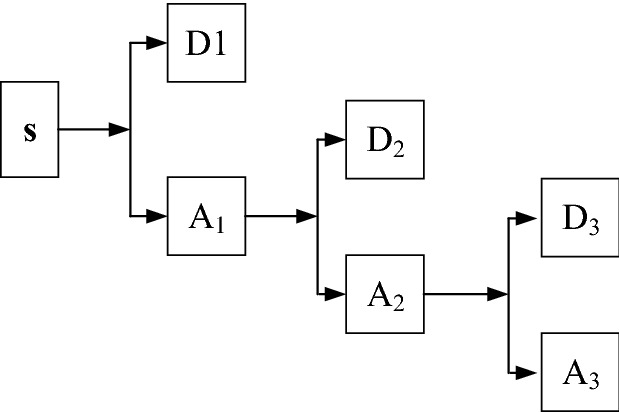
Three-level hierarchy chart of the DWT.

Specifically, the Haar wavelet is viewed as an effective initial wavelet function based on extensive numerical experiments, whose basis function *h*_*k*_(*z*) is described as^34^,

2 $$ h_{k} (z) = h_{{pq}} (z) = \frac{1}{{\sqrt N }}\left\{ {\begin{array}{*{20}l}    {2^{{{p \mathord{\left/ {\vphantom {p 2}} \right. \kern-\nulldelimiterspace} 2}}} } \hfill & {{{(q - 1)} \mathord{\left/ {\vphantom {{(q - 1)} {2^{p}  \le z \le {{(q - 0.5)} \mathord{\left/ {\vphantom {{(q - 0.5)} {2^{p} }}} \right. \kern-\nulldelimiterspace} {2^{p} }}}}} \right. \kern-\nulldelimiterspace} {2^{p}  \le z \le {{(q - 0.5)} \mathord{\left/ {\vphantom {{(q - 0.5)} {2^{p} }}} \right. \kern-\nulldelimiterspace} {2^{p} }}}}} \hfill  \\    {2^{{{p \mathord{\left/ {\vphantom {p 2}} \right. \kern-\nulldelimiterspace} 2}}} } \hfill & {{{(q - 0.5)} \mathord{\left/ {\vphantom {{(q - 0.5)} {2^{p}  \le z \le {q \mathord{\left/ {\vphantom {q {2^{p} }}} \right. \kern-\nulldelimiterspace} {2^{p} }}}}} \right. \kern-\nulldelimiterspace} {2^{p}  \le z \le {q \mathord{\left/ {\vphantom {q {2^{p} }}} \right. \kern-\nulldelimiterspace} {2^{p} }}}}} \hfill  \\    0 \hfill & {otherwise,z \in [0,1]} \hfill  \\   \end{array} } \right. $$

where *k* can be uniquely decomposed into *k* = 2*p* + *q*-1 and *h*_0_(*z*) is defined by $h_{0} (z) = h_{0,0} (z) = \frac{1}{\sqrt N },z \in [0,1]$. The wavelet decomposition coefficients obtained by mathematical derivations are further compared with a threshold and weighted to form a noiseless reconstruction of the signal. The wavelet decomposition follows the hierarchical rule in that a signal based on high-level decompositions can be factored into lower-level approximates. For illustration purposes, Fig. 1 shows the hierarchy chart of a multi-level wavelet decomposition, where A and D denotes the approximate and the detailed wavelet components, respectively. A comprehensive presentation of the DWT may be referred to^34^.

**Figure 2:**
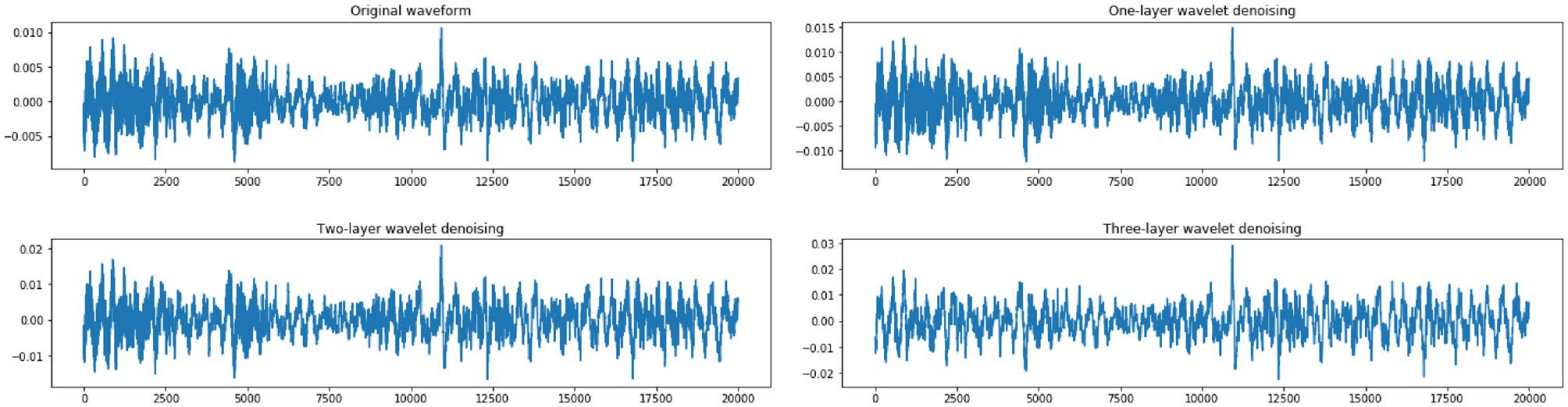
Signal denoising through a three-level Haar-based DWT.

In Fig. 2, we show a raw waveform and its denoised versions reconstructed by a three-level Haar-based DWT. It is observed that the DWT effectively reduces high-frequency noises while the trend of the signal is preserved very well.

### Spectrogram

The STFT is a process of computing the discrete Fourier transform (DFT) over short overlapping windows, and provides an analytical insight into correlations between the time-domain and the frequency-domain information of an acoustic signal. We perform the framing operation to divide the signal into a number of fixed-length clips and conduct the DFT on each clip interval. Small window lengths improve the temporal resolution of the STFT and enhance the capability to discriminate impulses that are closely spaced in time, however, at the expense of low frequency resolutions.

In this paper, we set the length of the windowed signal to 1024, and the number of samples between adjacent STFT columns (i.e., hop length) to 512 to obtain an efficient tradeoff between time and frequency resolutions. To optimize the effects of analyzing environmental sounds, it is preferable to adopt the frequency-domain representations in terms of Mel-scaled frequencies, which are nonlinearly related to physical frequencies and characterize the human auditory mechanism intuitively. Hence, we transform the frequency components obtained by the Fast Fourier Transform (FFT) over short-term windows into a bank of Mel-scaled band-pass filter banks. Assume that the filter bank has *M* triangular filters each with a center frequency *f*(*m*), 0 ≤ *m* < *M*, and these filters are of equal bandwidth in the Mel frequency range, the Mel frequency response of each filter is defined as^35^

3 $$  H_{m} (k) = \left\{ {\begin{array}{*{20}l}    {0,} \hfill & {k < f(m - 1)} \hfill  \\    {\frac{{k - f(m - 1)}}{{f(m) - f(m - 1)}},} \hfill & {f(m - 1) \le k \le f(m)} \hfill  \\    {\frac{{f(m + 1) - k}}{{f(m + 1) - f(m)}},} \hfill & {f(m) \le k \le f(m + 1)} \hfill  \\    {0,} \hfill & {k > f(m + 1)} \hfill  \\   \end{array} } \right.  $$

where *f*(*m*) is expressed as,

4 $$ f(m) = \left( {\frac{N}{{f_{s} }}} \right)F_{mel}^{ - 1} \left( {F_{mel} (f_{l} ) + m\frac{{F_{mel} (f_{h} ) - F_{mel} (f_{l} )}}{M + 1}} \right) $$

where *f*_*l*_ and *f*_*h*_ represent the lowest frequency and the highest frequency in the filter range, respectively, *N* is the length of the FFT, *f*_*s*_ denotes the sampling frequency, and *F*_*mel*_ is given by,

5 $$ F_{{{\text{mel}}}} = 1125\ln \left( {1 + \frac{f}{700}} \right) $$

**Figure 3:**
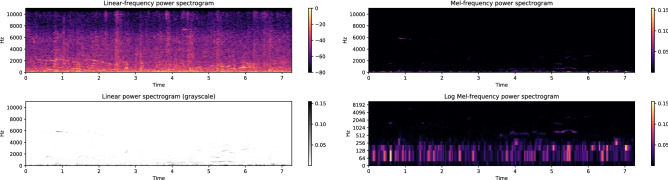
STFT spectrogram and Mel spectrogram.

In this paper, the frequency-domain analysis is performed by using a 20 ms Hamming window with 50% overlap and 1024 FFT bins. Triangular filters linearly spaced in the Mel scale are used to convert the STFT spectrogram into the Mel spectrogram. In Fig. 3, we present the STFT and the Mel spectrograms of an environmental sound signal over linear and logarithmic (log) frequency scales, respectively. The spectrograms, which are manifested as 2D heatmaps, show evidently correlations existent between time and frequency domains. Note that high-frequency components are effectively suppressed in the log-scale Mel spectrogram. The transformation from signal waveforms to heatmaps empowers us with the capability to leverage the vast amount of potentials manifested by CNN models, which were initially developed for image classification tasks.

**Figure 4:**
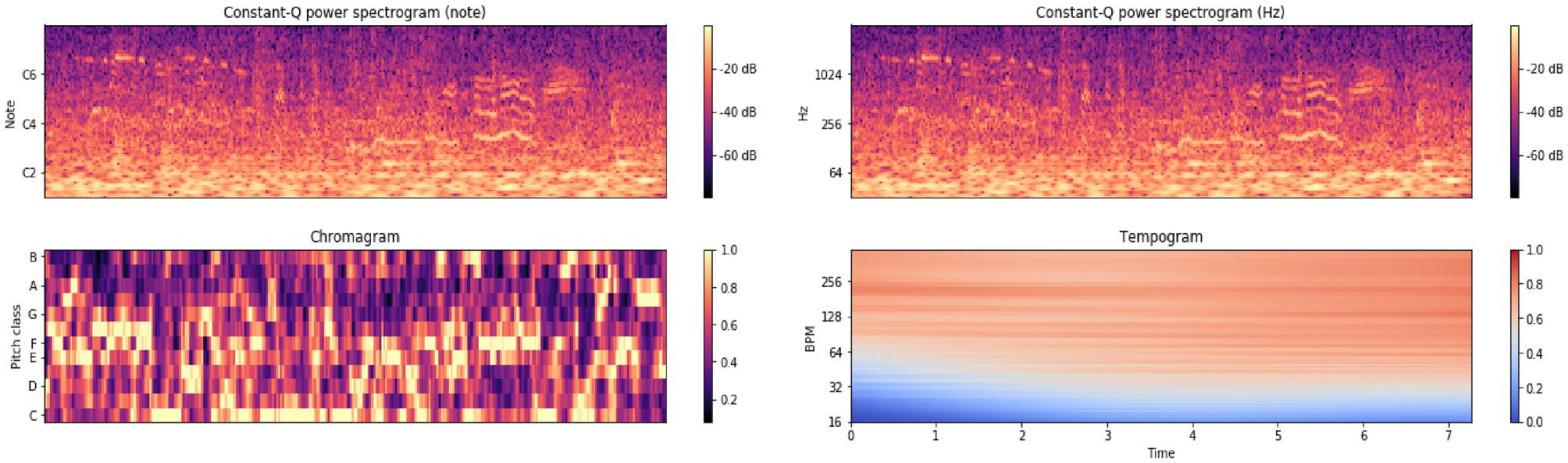
Chroma-CQT, chromatogram, and tempogram of an environmental sound signal, respectively.

In addition to the STFT spectrogram, we further derive other frequency-domain features based on the expert knowledge on the raw signal. Leveraging the representation diversities, we are capable of providing complimentary auditory information to assist in differentiating between complex acoustic scenes, e.g., chromatogram (Chroma), Constant-Q Chromagram (Chroma-CQT), Chroma-Energy Normalization (Chroma-CENS), and Tempogram based on the local autocorrelation of the onset strength envelope. In particular, the chromatogram is an efficient representation of the signal, which is frequently used in music genre analysis and projects the entire spectrogram onto 12 bins each representing a distinct semitone of the octave. Based on the chromatogram, we further extract uniform local binary pattern (LBP) textual descriptors, perform the normalization of each vector, quantize amplitudes based on pre-defined thresholds, and smooth the result with a sliding window. Through extensive numerical experiments, it is demonstrated that the chromatogram features are robust to acoustic dynamics and provide an intuitive approach to perform the gain control on signals that span multiple frequency bins. Hence, it is a desirable feature for the application of audio retrieval and particularly for the task of discerning signals that are subtly different. In Fig. 4, we show the chromatogram, Chroma-CQT and tempogram of an environmental sound signal, respectively. Compared with the Mel spectrogram, the chromatogram enables us to evaluate perceptually a sound signal without resorting to physical frequencies in that music notes that are one octave apart are deemed similar. These characteristics allow the model to effectively simulate the auditory performance by focusing on most conspicuous parts of the signal.

### Data augmentation

It is well known that the performance of deep-learning models is heavily limited by the size of training datasets. Seeking to enhance the generalization capacity of the model, we conduct extensive augmentations on training signals prior to constructing frequency-domain spectrograms. Conventional time-domain augmentation approaches include random time-shifting, Gaussian distributed or colored noise injection, amplitude adjustment, and speed/pitch moderation.

**Figure 5:**
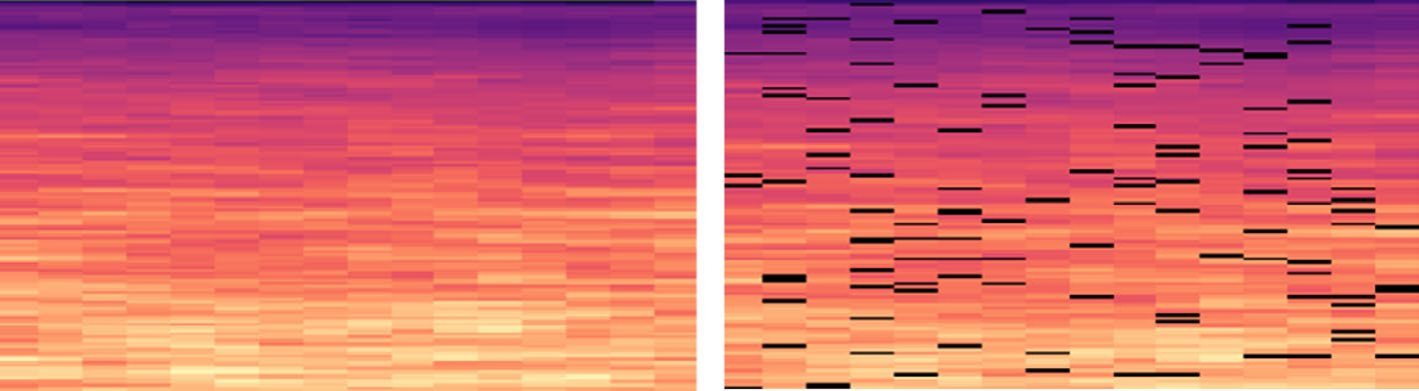
Coarse drop-out image-level augmentation applied to the Chroma-CQT.

A unique feature of environmental sounds is that signals are often overlapped with each other. For instance, a sound signal recorded in a supermarket consists in multiple acoustic components and surrounding noises. In this paper, we propose to apply a frequency-domain augmentation method known as mix-up^36, 48^ to manipulate 2D spectrograms in order to simulate such overlapping effects. The mix-up approach uses a randomly generated beta-distributed parameter *λ* to combine two samples in the training data to generate a sample that is previously non-existent in the original data set. The technique takes into considerations linear expressions between training samples and significantly improves the representation capacity as well as the robustness of the model. The method of generating virtual samples is formulated by,

6 $$ \mu (\tilde{x},\tilde{y}|xi,yi) = \frac{1}{n}\sum\nolimits_{j}^{n} {\mathop E\limits_{\lambda } \left[ {\delta (\tilde{x} = \lambda x_{i} + (1 - \lambda )x_{j} ,\tilde{y} = \lambda y_{i} + (1 - \lambda )y_{j} )} \right]} $$

where *λ* ~ Beta (*α*, *α*), *α* $\in$(0, ∞) and $\widetilde{x}$, $\widetilde{y}$ are formulated as:

7 $$ \left\{ \begin{gathered} \tilde{x} = \lambda x_{i} + (1 - \lambda )x_{j} \hfill \\ \tilde{y} = \lambda y_{i} + (1 - \lambda )y_{i} \hfill \\ \end{gathered} \right. $$

where *x*_*i*_ and *x*_*j*_ denote raw input vectors, *y*_*i*_ and *y*_*j*_ are one-hot label encodings, and (*x*_*i*_, *y*_*i*_) and (*x*_*j*_, *y*_*j*_) represent two samples taken randomly from the training data. Following the operations of scaling pixel values to the range of 0 to 255, spectrograms can be equivalently viewed as a three-channel red–green–blue (RGB) image. Furthermore, we apply a number of pixel-level image transformations to enhance the diversity of the training data. The drop-out operation as shown in Fig. 5 can be used to simulate the scenario where the signal has no certain frequency components by randomly masking a small percentage of pixels, i.e., setting their values to zeros.

## Multi-input 3D CNN model

In recent years, CNN models have become a predominant solution in classification, regression and text processing tasks. The advantages of CNN models are attributed to a combination of properties including the shared kernel weights and the adjustable network depth. The dimension of the kernel is related to the convolution window size and the number of output feature maps. Another key component is the nonlinear activation function, which takes the format of the rectified linear unit (RELU) or a leaky RELU function to activate the subsequent layers of network nodes. Moreover, various regularization operations including batch, instance, and spectral normalizations facilitates the training of deep learning models, while pooling operations allow the model to learn position invariances over local regions. Given a specified loss function, the backward-propagation (BP) algorithm is invoked to update the weights of each layer in a progressive manner. The proposed model is based on 3D convolutions, which captures temporal dependencies existent in audio clips more effectively as compared with 2D convolutions by performing the operation on *a stack of* spectrograms obtained from the different perspectives of the signal. Hence the proposed model is able to obtain a sequence of learned features and exploit both long-term and short-term correlations inherent in environmental sounds. The advantages of 3D models over 2D models are demonstrated in^37–39^ in video processing applications including the detection of spatial geometries and video action recognitions.

**Figure 6:**
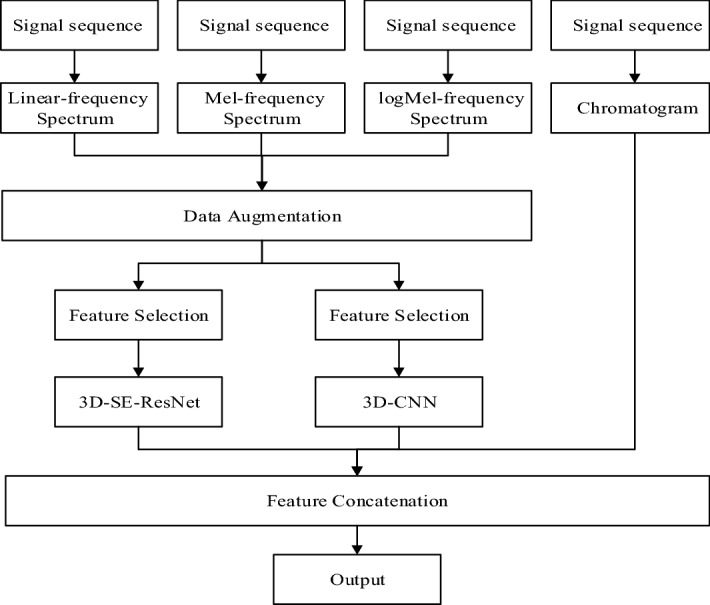
Flowchart of the proposed multi-input 3D model for environmental sound classification.

**Figure 7:**
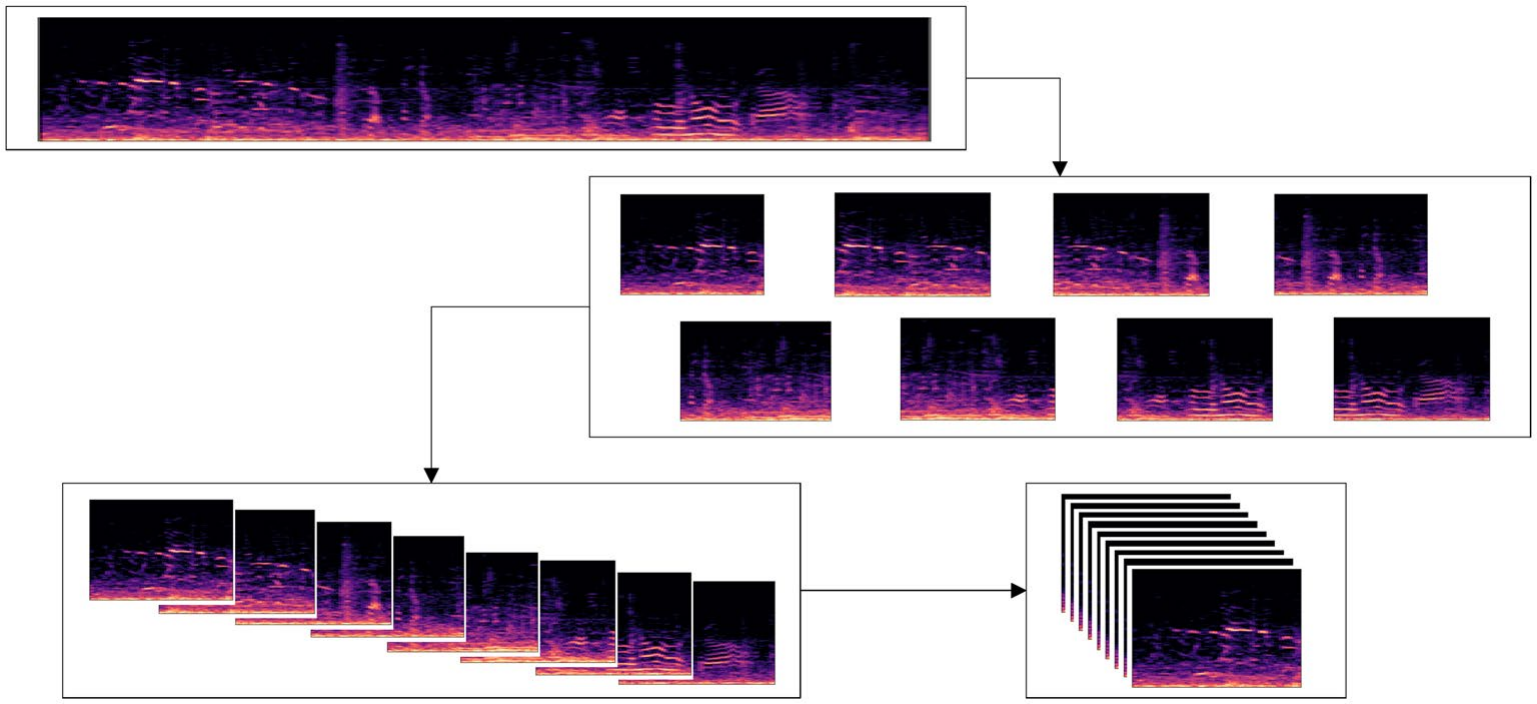
Construction of 3D Feature Maps at the input to the model.

In this paper, we split a 10-s audio signal into four 4-s clips with 50% overlapping between clips. In order to avoid information loss and fully exploit information provided by various signal presentations, we use three types of spectrograms, i.e., the STFT spectrogram, the Mel spectrogram and their first-order derivatives to construct multi-channel features of each clip. The spectrograms of all clips are stacked to form a four-dimensional (4D) input to the proposed model in the format of [*t*, *h*, *w*, *c*], where *t* = 4 is the number of clips, *h* and *w* denote the height and width, and *c* = 3 denotes the number of channels corresponding to the above-mentioned three types of spectrograms, respectively. By stacking multiple layers of 3D convolution kernels, the proposed model enhances the learning of spatial–temporal representations across clips. Furthermore, we form the chromatogram directly based on raw waveforms, which represents pitch and note information used in audio matching applications, and concatenate the chromatogram statistics with deep features learned from the final 3D convolution layer before forwarding to the classification layers. The derivation of multi-domain features enables the model to learn comprehensive acoustic characteristics obtained at various levels^49^. In addition, the chromatogram serves a regularization purpose by supplementing deep features with auxiliary shallow-level information. In Fig. 6, we present the flowchart of the proposed architecture, which itself consists of two sub-branches, i.e., a sequential 3D branch and a 3D SE-ResNet branch. For clarity purposes, we also present in Fig. 7 the flowchart of constructing 3D feature maps at the input to the proposed model. The input signal is partitioned into short audio clips based on which a 3D cube of spectrograms can be obtained by stacking vertically the spectrogram of each clip.

**Figure 8:**
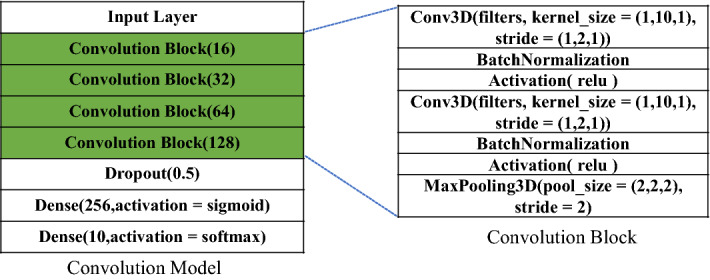
3D Sequential Branch in the proposed architecture.

Figure 6 shows that the proposed architecture consists of two branches of 3D CNN blocks. The augmented spectrograms are taken as the input to the sequential 3D branch. The feature selection block in Fig. 6 represents the normalization and scaling operations to ensure that the pixel values are in the range of 0 to 255 as well as an operation to regularize pixel values, i.e., set pixels with values lower than a threshold (e.g., 15) to zero. The resulting input to the model is thus a stack of three-channel RGB-like images and can be directly used as the 3D CNN model input. The 3D branch consists of a sequence of 4 blocks with an increasing number of feature maps ranging from 16 to 128. Each block is composed of two 3 × 3 × 3 convolution layers, each of which is followed by a max-pooling operation to reduce the resolution by half, and a batch normalization layer for regularization purposes. The input to each CNN layer is padded with zeros to ensure that the dimension of the output remains the same as the input. With the dimension of feature maps gradually reduced, we increase the number of channels seeking to preserve a sufficiently representative feature at deep layers. Figure 8 shows the detailed structure of the sequential 3D branch in the proposed architecture.

**Figure 9:**
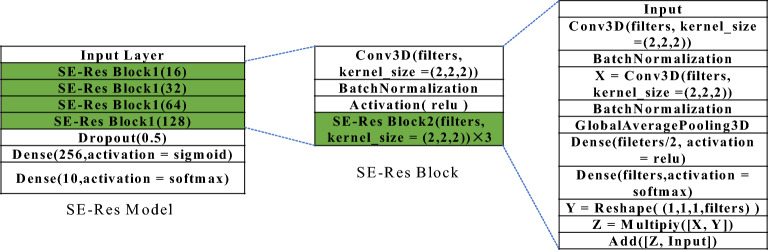
3D SE-ResNet branch in the proposed architecture.

Inspired by the ResNet, we modified the sequential branch to construct an additional branch by introducing a residual connection inside each convolution block, which forces the model to fit on a residual mapping of the input. Hence the objective function approximates the identity mapping and enables the network to learn discriminant features in a robust manner^40^. Figure 9 shows the block diagram of the 3D ResNet branch. As shown in Fig. 9, the residual block itself consists of two 3D convolution layers with a skip connection bridging the input of the first layer and the output of the second layer by introducing a summation operation. Such “short direct connections” allows the model to learn directly from shallow-level features and avoid the gradient degradation problem without incurring additional parameters. To enable the model to focus on the attentive part of the spectrogram, we introduce the squeeze-and-excitation (SE) module^41^ as an attention mechanism in the residual block. The SE Block redistributes the importance of data across different channels by performing the squeeze, excitation and scale operations sequentially. In the squeeze stage, we compress the global information of each channel into a 1D descriptor, which may be viewed as a weighting factor. In the excitation stage, a gating mechanism with the sigmoid activation is employed to fully capture channel-wise dependencies, based on which the model becomes more flexible and is capable of learning effectively nonlinear interactions between various channel components. Subsequently, the weights are multiplied with the output of the residual block and attached to the derived features per channel. The SE operation enhances the feature-learning capabilities of the 3D model to effectively grasp hierarchically discriminant attributes at various convolution layers.

## Numerical experiments

### Dataset

The DCASE competition was launched by the University of London Queen Mary College Digital Music Center and Institut de Recherche et Coordination Acoustique/ Musique (IRCAM) in 2013. Currently, it is one of the most recognized competitions in the field of acoustic event detection and classification. The DCASE datasets are extensively used as a performance benchmark of various algorithms as the competition typically features a large amount of training data.

**Figure 10:**
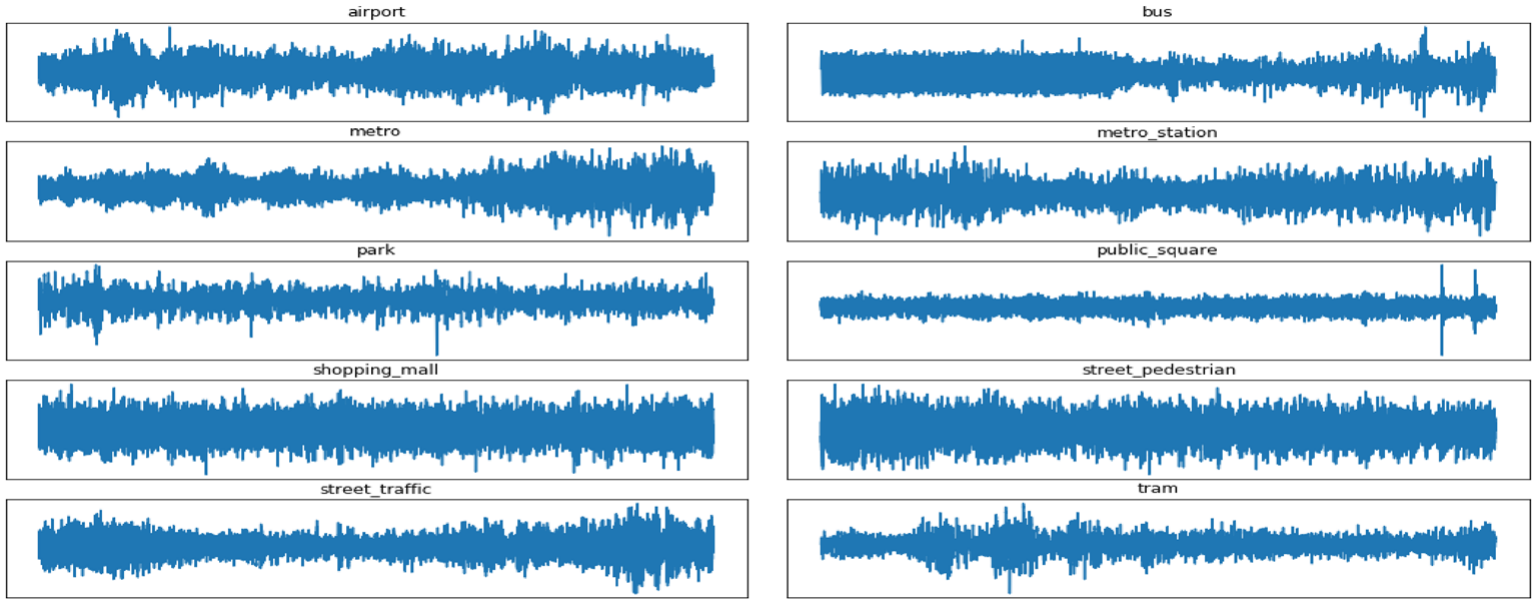
Signal waveform of each category in the DCASE2019 dataset.

In this paper, we perform numerical experiments on the dataset of the Sound Scene Detection and Classification Sub-Task of the DCASE 2019 competition. The development data set includes audio recordings from 10 cities at the sampling rate of 44.1 kHz. The dataset covers a total of 10 scene categories, including Airport, Bus, Metro, Metro Station, Park, Public Square, Shopping Mall, Street Pedestrian, Street Traffic and Tram. It contains 40 h of training data and a total of 14,400 samples (each sound scene category has 144 samples as recorded in one city), with the duration of each sample set to 10 s. Figure 10 presents the raw waveforms of the ten sound scene categories.

### Results

In the experiment, we divide the development data into training and validation subsets according to the ratio of 80:20. The validation set is designated to be independent of the training process and used only as a performance indicator. In order to objectively evaluate the performance of the model, we ensure that the validation set and the training set are strictly non-overlapped with each other. To alleviate overfitting risks, we perform extensive data augmentations on raw signals including both time-domain and spectrogram transformations. For mix-up operations, we set the mix-up ratio to be a random number chosen from 0 to 0.05, which achieves a tradeoff between the convergence behavior and the model classification performance. For the purpose of achieving low computational complexities, the signal sampling rate is lowered to 16 kHz as compared to the original 44.1 kHz, based on which we extract the STFT spectrogram, the Mel spectrogram, and the chromatogram as the input to the model. To ensure a fair comparison with other algorithms in the literature, we use five-fold cross-validations (CV) to evaluate the performance of the proposed model. As shown in Fig. 6, the proposed model aggregates deep features learned from the sequential 3D branch and the 3D SE-ResNet branch, and subsequently concatenates with the statistical characteristics of the chromatogram derived directly from the input signal.

**Figure 11:**
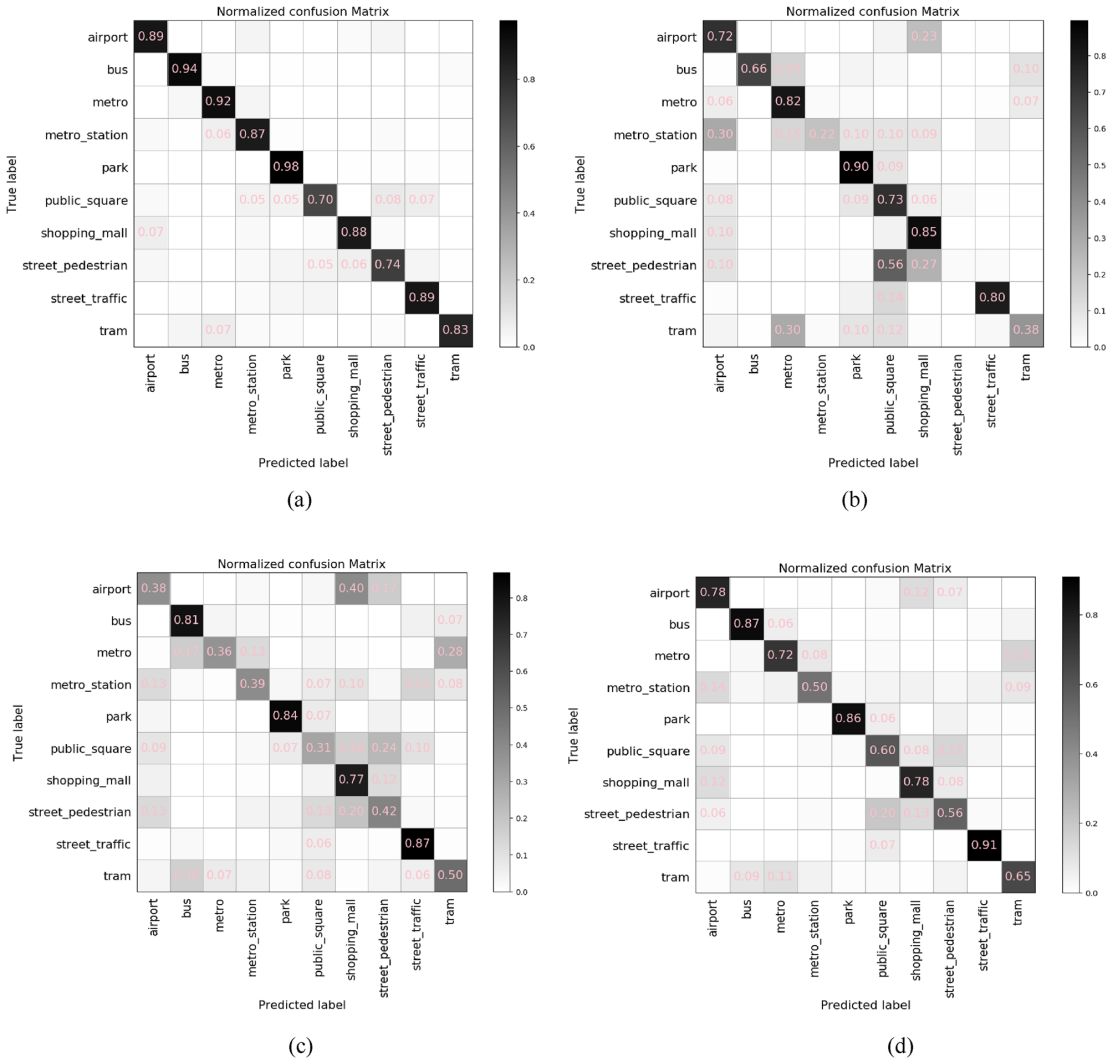
Performance comparison of the proposed approach with other models in terms of confusion matrices. (**a**) Proposed 3D CNN model. (**b**) 1D sequential model. (**c**) 2D ResNet 50 model. (**d**) 2D sequential model.

The performance of the proposed model can be visually represented through a confusion matrix evaluated on the validation data, which can be divided into 10-class data as shown in Fig. 11. The confusion matrix is a specific table layout that allows direct visualization of the performance in each class. It reports the errors and confusions among different classes by calculating the correct and incorrect classification for each class and accumulating the results in the table. The horizontal axis represents the predicted samples and the vertical axis represents the true samples. The probabilities of correctly classified results are recorded on the diagonal, while those of incorrect predictions are scattered through the matrix. In Fig. 11a, we present the normalized confusion matrix of the proposed 3D model indicating the classification accuracy on each class and the probability of mis-classification into the other classes. The average accuracy of the five-fold cross-validation is 86.4%. In several scenarios such as Airport, Bus, Park, and Shopping Mall, the model achieves excellent performance and classifies signals accurately. In the scenarios such as Public Square and Street Pedestrian that are very likely to be confused with each other, the classification accuracy of the model is lowered to 75%. Based on the literature^42^, the human auditory system is susceptible to mis-classifying mixed environmental sounds arising simultaneously from heterogeneous sources with an average accuracy of 75%. Hence, the proposed model achieves impressive performance and succeeds in obtaining human-level performance in discerning complicated sound scenes by exploiting a substantial number of multi-domain features.

For comparison purposes, we also present the performance of the 1D sequential model, the 2D ResNet50 model, and the 2D sequential CNN model in Fig. 11, which achieves the five-fold accuracies of 56.6%, 72.3% and 60.1%, respectively. The 1D model accepts the raw signal waveforms as the input with an optimized large kernel size of 19 applied at the first convolution layer seeking to capture global temporal patterns. Since environmental sound are typically distorted by various noise components, the 1D model show a low accuracy of 38% when differentiating tram from other classes. The 2D sequential model shows a noticeable performance improvement, which generates the frequency-domain spectrogram based on 10-s signals. The spectrogram enables the model to learn discriminant deep features by exploiting the relations between temporal and frequency domains. It is observed that the model architecture shows an impact on the final classification performance. The 2D ResNet50 model improves the accuracy to 72.3% by employing residual connections between convolution blocks, thus facilitating the information flow and avoiding the problem of gradient diminish in the training process. However, numerical experiments show that further increasing the network depth has little effect on the performance. On the contrary, the proposed approach obtains the state-of-the-art performance of 86.4% by incorporating two 3D sub-branches with different depths and widths to extract supplementary features from a combination of spectrogram representations. Moreover, the feature-fusion mechanism, which combines the learned deep features with the shallow chromatogram statistics, enables the model to achieve better performance as compared with a single feature extractor.

In Table 1, we compare the performance of our method with several advanced models in the literature on the validation set. On contrast to most models that employ the ensembling of several models at the cost of high computational complexities, the proposed approach is a single end-to-end deep-learning model. For instance, Kong^23^ implemented three CNN models of various depths, i.e., 5-layer, 9-layer and 13-layer, respectively, and numerically averaged the results of three models. In^43^, an ensembling method to integrate the results of eight classifiers was presented. In^44^, a multi-input model based on a spectrogram decomposition approach was proposed and numerical averaging is further invoked to improve performance. On the other hand, our model avoids the cost to train multiple models as in^45^. Furthermore, the model is capable of achieving high spatial–temporal resolutions in discerning acoustic signals by incorporating 3D convolutions, which possesses the inherent advantage of learning simultaneously the temporal and spatial features in the input signals. The attention mechanism further empowers the model to focus on the most conspicuous part of the learned features and enhances the convergence of the training process.

**Table 1:** Classification accuracy of various methods.

Method	Data augmentation	Features extraction	Sample rate (kHz)	Accuracy (%)
CNNs(5/9/13)^23^	\	Mel energies	32	69.2
ResNet^43^	Mix-up	HPSS	48	71.9
CNNs_Averaging8^44^	\	HRTF, NNF	44.1	64.0
CDNN^46^	Mix-up	Single frequency cepstral coefficients (SFCC), log-Mel energies	48	70.4
3D-SEResNet	Mix-up	Mel Spectrogram	16	80.1
3D-SEResNet	Mix-up	Log-Mel Spectrogram	16	81.3
3D-CNN	Mix-up	STFT	16	83.5
Our Method	Mix-up	Mel Spectrogram, Chromagram, STFT	16	86.4

In Table 1, we compare the performance of the proposed 3D model with several 2D schemes in the literature. It is shown that the proposed model achieves noticeable performance gains even at lower signal sampling rates. Moreover, the data augmentation strategies of mix-up and coarse drop-out on the frequency-domain spectrograms produce significant improvements, which may be attributed to the fact that these operations generate simulated samples that are highly similar to those in the actual recoding environments. In Table 1, we also include a comparison between various architectures of 3D CNNs, i.e., the proposed approach, 3D sequential CNN, and 3D SeResNet using different scales of Mel spectrograms. It is shown that the proposed approach, which combines complementary representations of signal features, achieves much better performance over those based on single feature extraction. The fusion network, which concatenates chromatogram statistics and deep convolution features, effectively alleviates the problem of information loss when the input signal is propagated through 3D CNN models by using domain-specific knowledge to extract supplementary shallow features. Based on the proposed method, we are capable of discerning accurately complex sound scenes that feature signals from multiple sources and are mixed up with background noises by taking the end-to-end data-centric approach and fully exploiting the advantages provided by various network architectures.

## Conclusion

Motived by the advantages of 3D convolutions in the area of video action recognitions, in this paper we propose a novel multi-input multi-branch 3D CNN model that accepts the STFT spectrogram and the Mel spectrogram as the input to derive deep convolution features, which are subsequently concatenated with the statistics of the chromatogram drawn directly from input signals. The proposed model incorporates attention mechanisms and uses extensive data augmentations to increase the diversity of training data. The novelties of constructing the multi-domain spectrograms and conducting deep and shallow-level feature fusion effectively improve the capability of the proposed model in the task of environmental sound classification. Numerical results on a large dataset show that the proposed model achieves noticeably higher classification accuracies and demonstrates impressive human-level performance in discerning complex acoustic scenes.
